# CSF profile in primary progressive multiple sclerosis: Re-exploring the basics

**DOI:** 10.1371/journal.pone.0182647

**Published:** 2017-08-10

**Authors:** Ahmed Abdelhak, Tilman Hottenrott, Christoph Mayer, Gudrun Hintereder, Uwe K. Zettl, Oliver Stich, Hayrettin Tumani

**Affiliations:** 1 Department of Neurology, Ulm University, Ulm, Germany; 2 Department of Neurology, University Hospital Freiburg, Freiburg, Germany; 3 Department of Neurology, University Hospital Frankfurt, Frankfurt, Germany; 4 Department of Neurology, Neuroimmunological Section, University Hospital Rostock, Rostock, Germany; Heinrich-Heine-Universitat Dusseldorf, GERMANY

## Abstract

**Objective:**

The aim of this study was to report the basic cerebrospinal fluid (CSF) profile in patients with primary progressive multiple sclerosis (PPMS).

**Methods:**

The results of CSF analysis from 254 patients with PPMS were collected at four university hospitals in Germany. Routine CSF parameters and different indices of intrathecal immunoglobulin synthesis were evaluated. We assessed possible correlations between the various CSF parameters and the expanded disability status scale (EDSS) both at the time of lumbar puncture and during the course of the disease.

**Results:**

The median cell count and albumin concentration in the CSF did not deviate from normal values. The CSF-serum albumin-quotient (Q_ALB_) was elevated in 29.6% of the patients, while intrathecal immunoglobulin G (IgG) oligoclonal bands (OCBs) were detected in 91.1% of the patients. CSF-lactate levels as well as local IgM- and IgA-synthesis were correlated with the yearly disease progression rate, as assessed by EDSS.

**Conclusion:**

We present the results of the hitherto largest and most detailed CSF biomarker profile in a cohort of 254 patients with PPMS. As reported previously, OCBs are the most sensitive marker for intrathecal IgG synthesis. CSF-lactate concentrations are positively correlated with the progression rate, which might suggest that mitochondrial dysfunction plays a relevant role in PPMS. The negative correlation between intrathecally produced IgM and IgA and disease progression may indicate their hitherto unexplored protective role.

## Introduction

Primary progressive multiple sclerosis (PPMS) is currently considered an entity in multiple sclerosis (MS) disease spectrum, representing about 15% of MS patients[1]. However, PPMS patients differ in many clinical, pathological, and imaging aspects, which explains the necessity of various diagnostic guidelines[2]. Indeed, the diagnostic guidelines for PPMS have been revised over the last few years[3–6]. In contrast to relapsing-remitting multiple sclerosis (RRMS), the cerebrospinal fluid (CSF) examination remains a part of the McDonald diagnostic criteria[6]. At present, many studies addressing the CSF profiles of various MS subtypes have been published. However, these studies had obvious limitations, including a relatively small number of PPMS patients (only one study reported data from more than 100 PPMS patients) as well as limited CSF parameter datasets[7–9]. Therefore, we initiated a multicenter study to systematically collect and analyze detailed CSF profiles containing all parameters commonly assessed in clinical practice in a well-characterized PPMS cohort consisting of more than 250 patients.

## Methods

### Data collection

CSF data were collected from four university hospitals in Germany (Ulm, Frankfurt, Rostock, and Freiburg). We included PPMS in- and outpatients treated between 2010 and 2015. Each PPMS diagnosis was established according to the 2010 revisions of the McDonald criteria[6] after careful exclusion of relevant differential diagnoses. Lumbar puncture (LP) was performed for diagnostic purposes only with the written consent of all patients. CSF and serum samples were taken on the same day and stored according to consensus protocol for the standardization of CSF collection and biobanking[10]. Records of all available patients matching these criteria were retrospectively reviewed regarding age at onset, initial neurological complaints, age at first diagnosis, time between clinical onset and diagnosis, expanded disability status scale (EDSS) at the time of LP (EDSS_LP_), EDSS at the last documented follow-up (EDSS_FU_), and treatments. Age at clinical onset and initial complaints were obtained from the available medical records and assessed according to the first documented neurological symptoms attributable to the disease. We divided the initial complaints into four main categories: motor, sensory, cerebellar, and other. The “other” category comprised brain stem syndromes, visual disturbances, cognitive symptoms, complex partial seizures, and bladder dysfunction. The clinical severity of MS was assessed using the EDSS score[11] and determined by a certified EDSS rater. We calculated the yearly progression rate by dividing the EDSS_FU_ over the period between clinical onset and date of the EDSS_FU_.

CSF analysis included basic parameters, such as the total cell count; the CSF-serum quotient for albumin (Q_ALB_); quotients of immunoglobulin G, M, and A (IgG, IgM, and IgA); CSF-lactate concentration; oligoclonal bands (OCB) pattern[9]; and measles, rubella and zoster (MRZ) reaction[12]. In cases of repeated LP for the same patient, only the results of the LP used to establish the diagnosis were analyzed. Q_ALB_ was used as an indicator for the blood-CSF barrier (BCB) function[13], and it was assessed according to the age-related reference range (4+ age/15)[14]. The IgX index was calculated using the following formula: (IgX _CSF_ / IgX _Serum_: Albumin _CSF_ / Albumin _Serum_)[15]. IgG-index values > 0.7 [16], IgM-index values > 0.061 [17], and IgA-index values > 0.34 [18] were considered elevated. The concentration of intrathecal IgG, IgM, and IgA -synthesis (IgG_loc_, IgM_loc_, and IgA_loc_) was calculated according to the following formula: IgX_loc_ = (QIgX—Q_lim_). IgXserum. Following Reiber, the formula for Q_lim_ was Q_lim_ = a/b √Q_ALB_^2^ + b^2^ –c, where a/b, b^2^, and c values differ for each immunoglobulin type[19]. Negative values were reported as zero. IgG OCBs were classified into the following five patterns[20]: no bands detected in CSF or serum (pattern 1), bands detected in CSF only (pattern 2), bands detected in CSF *plus* additional identical bands in CSF and serum (pattern 3), identical bands detected in CSF and serum (pattern 4), and monoclonal bands detected in CSF and serum (pattern 5).

We considered the MRZ reaction to be positive when the reported antibody index (AI) against at least two of the included indices was > 1.4 [21].

The patients were further classified into three categories. PPMS_T_ included patients who received medical treatment over the disease course; PPMS_TN_ included treatment-naïve patients, and LP_D_ included those who received diagnostic LP before any medical treatment began.

### Statistics

All statistical tests were performed using IBM SPSS Statistics, version 21 (Armonk, USA). We used the Shapiro–Wilk test to examine the distribution of the data and the Mann–Whitney U test or the Kruskal–Wallis test to compare the medians of different variables. Fisher’s exact test was used for qualitative variants, and Spearman’s rho test was used to measure correlation. A p-value < 0.05 was considered statistically significant. No Bonferroni correction was done due to the exploratory nature of this study.

### Protocol approval

The study was reviewed by the appropriate ethics committee of the University of Ulm (approval number 20/10) and was performed in accordance with the ethical standards the 1964 Declaration of Helsinki. Written informed consent for the LP was obtained from all patients participating in this study.

## Results

### Clinical characteristics of the patients

**Fig 1:**
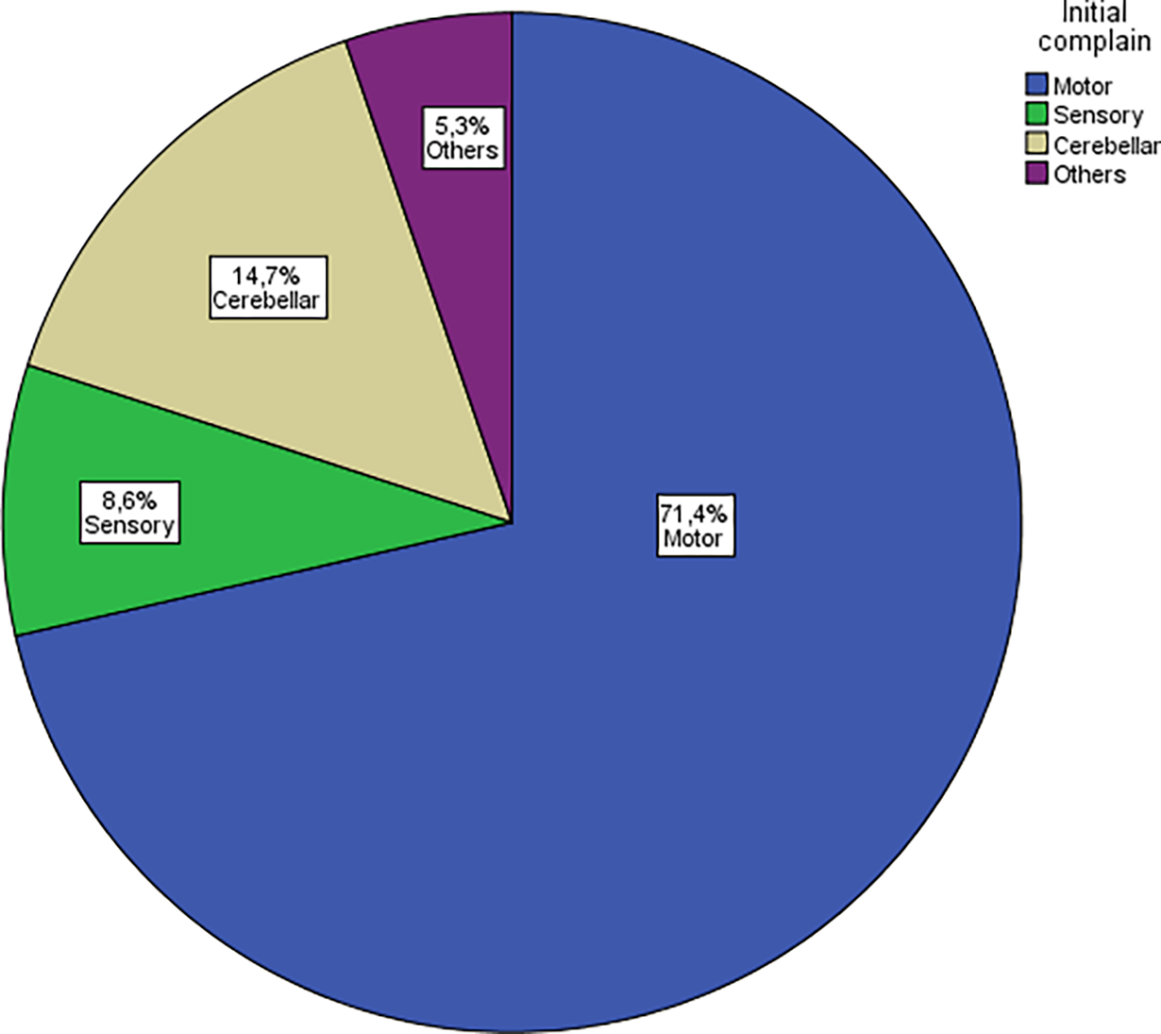
Distribution of the initial manifestations. Motor weakness was the most common initial symptom in about 71.4% (175/245) of patients included in our multicenter cohort, followed by cerebellar manifestations in 14.7% (36/245) and sensory disturbances in 8.6% (21/245). Other initial manifestations such as brain stem syndromes, visual disturbances, cognitive symptoms, complex focal seizures or urinary disturbances were present in 6.3% (13/245).

The clinical features of the 254 enrolled PPMS patients are summarized in **Table 1**and **Fig 1**. Overall, 154 patients received one or more of the following therapies over the course of the disease: 3-month pulse steroid therapy (n = 71), mitoxantrone (n = 29), cyclosporine (n = 1), rituximab (n = 6), intravenous immunoglobulins (n = 1), dimethyl fumarate (n = 1), interferon beta-1a (n = 2), glatiramer acetate (n = 1), azathioprine (n = 4), and more than one therapy (n = 38). However, we did not include details such as the duration of the therapies because this was not the focus of the study. It should be mentioned that none of these therapies is approved for PPMS.

**Table 1:** Clinical features of primary progressive multiple sclerosis (PPMS) patients.

Clinical features of primary progressive multiple sclerosis (PPMS) patients (median, interquartile range)	
**Number**	254
**Gender (male: female)**	1: 1.1
**Age at first symptom**	44 (37–51)
**Age at diagnosis**	47 (41–55)
**Time between first symptom and diagnosis in years**	3 (1–5)
**Expanded disability status scale (EDSS) at the date of lumbar puncture (LP)**	4.0 (3.0–5.5)
**Follow up period in months**	27 (1–74)
**EDSS at follow-up**	6.0 (4.0–6.5)

Patients with a motor symptom at clinical onset (PPMS_Motor_) scored higher on the EDSS_LP_ compared to patients with cerebellar manifestations (PPMS_Cerebellar_) or patients with sensory symptoms (PPMS_Sensory_) (4.5 vs 3.5 or 3.5, p < 0.001, n = 201). The yearly progression rate did not differ between these three subgroups with different initial symptoms (p = 0.3). Similarly, analysis of PPMS_TN_ revealed a higher EDSS_LP_ in the PPMS_Motor_ group compared to the PPMS_Cerebellar_ and PPMS_Sensory_ groups (4.5 vs 3.5 vs 4.0, p = 0.03). The yearly progression rate did not differ between the groups (p = 0.2).

### CSF profile in PPMS

In some patients, clinical or CSF data were not obtainable from the records or could not be retrospectively evaluated. A summary of these results is provided in **Table 2**.

**Table 2:** Summary of the results of lumbar puncture (LP) in patients with primary progressive multiple sclerosis (PPMS).

CSF parameter	Median (maximum)		interquartile range		Missing data	
	Total PPMS cohort	LP_D_	Total PPMS cohort	LP_D_	Total PPMS cohort (total number = 254)	LP_D_ (total number = 209)
**Cell count (/μl)**	2 (101)	3 (101)	1–5	1–5	20	10
**Total protein (mg/l)**	485 (1775)	485 (1775)	181–323	366–602	21	10
**Albumin (mg/l)**	252 (1300)	247 (1300)	374–593	176–332	78	63
**Alb-Q (x10**^**3**^**)%**	5.8 (37.8)	5.8 (37.8)	4.3-7-8	4.2–7.9	27	17
**Lactat (mmol/l)**	1.7 (5.6)	1.7 (2.9)	1.5–1.9	1.5–1.9	153	127
**IgG index**	0.7 (6.0)	0.8 (6.0)	0.6–1.10	0.6–1.1	75	65
**IgG**_**loc**_	2.70 (271.7)	3.3 (271.7)	0–24.4	0–24.1	75	65
**IgA index**	0.3 (2.3)	0.3 (2.1)	0.2–0.3	0.3–0.3	78	68
**IgA**_**loc**_	0 (72.7)	0 (72.7)	0	0	78	68
**IgM index**	0.08 (0.97)	0.08 (0.97)	0.05–0.13	0.05–0.2	78	68
**IgM**_**loc**_	0 (4.5)	0 (2.5)	0	0	78	68

Loc = local synthesis in mg/dl, LP_D_: diagnostic lumbar puncture.

#### Cell count, total protein, albumin, and lactate

The cell count was elevated in 28.6% (n = 67) of patients. CSF-lactate levels were not elevated with a median of 1.7 mmol/L. The age-matched Q_ALB_ was elevated in 29.6% (n = 67/226) of the patients.

Similar results were found for the LP_D_ subgroup, in which 28.6% (n = 57) of patients had an elevated cell count. The CSF-lactate median was 1.7 mmol/L (0.5–2.9, n = 63), and the Q_ALB_ was elevated in 32.7% of the patients (n = 48/147).

#### Intrathecal immunoglobulin synthesis

Pattern 1 was found in 8.2% of the patients (n = 18/220). With 78.6%, pattern 2 was observed most commonly in our patients (n = 173/220) whereas pattern 3 was found in 12.3% of the patients (n = 27/220). Two cases showed pattern 4 OCBs.

The MRZ reaction was positive in 77 of 148 patients (52%). Patients having a biphasic reaction (only 2 AI are positive) constituted 25% (37/148) of patients with positive MRZ-Reaction. A triphasic positive reaction (M+,R+ and V+) was found in 27.0% (n = 40/148), whereas a monophasic positive reaction (M+ or R+ or V+) was detected in 31.1% (n = 46/148). The Prevalence of positive AI against single virus and of the various combination is mentioned in **Table 3**.

**Table 3:** Prevalence of single positive AI against measles (M), Rubella (R) and varicella-zoster virus (Z) and different combinations in our PPMS cohort.

Positive Antibody index	Measles (M)	Rubella (R)	Varicella Zoster (Z)	M+R+Z-	M+R-Z+	M-R+Z+	M+R+Z+	Positive MRZ-reaction (≥ two AI > 1.4)
**Total PPMS cohort**	24/148 (16.2%)	7/148 (4.7%)	15/148 (10.1%)	13/148 (8.8%)	13/148 (8.8%)	11/148 (7.4%)	40/148 (27.0%)	77/148 (52.0%)
**OCB+**	24/139 (17.3%)	7/139 (5.0%)	14/139 (10.1%)	13/139 (9.4%)	12/139 (8.6%)	11/139 (7.9%)	35/139 (25.2%)	71/139 (51.1%)
**OCB-**	0/9	0/9	1/9 (11.1%)	0/9	1/9 (11.1%)	0/9	5/9 (55.6%)	6/9 (66.7%)
**LP**_**D**_	18/127 (14.2%)	7/127 (5.5%)	13/127 (10.2%)	11/127 (8.7%)	12/127 (9.4%)	11/127 (8.7%)	34/127 (26.8%)	68/127 (53.5%)

OCB+: patients with positive oligoclonal bands, OCB-: patients with negative oligoclonal bands, LP_D_: diagnostic lumbar puncture.

The IgG-index values were elevated in 49.2% (n = 88/179), and IgG_loc_ tested positive in 61.7%. The IgM index was elevated in 58.5% (n = 103/176), and the IgM_loc_ was positive in 21.0% (n = 37/176). While the IgA index was elevated in 24.4% (n = 43/176), IgA_loc_ was detectable in 17.6% of the patients (n = 31/176).

Analysis of the LP_D_ subgroup revealed similar results. The IgG-index values were elevated in 51.4% (n = 74/144), while IgG_loc_ tested positive in 63.1% of this subgroup.

### Correlations between CSF findings and clinical parameters

#### Q_ALB_

Comparing the analysis of the whole group with that of the LP_D_ subgroup revealed no differences between the median EDSS_LP_ and median yearly progression rate, which were 4.0 and 0.58 in both groups, respectively, in patients with normal and elevated Q_ALB_. In the PPMS_TN_ subgroup, the median yearly progression rates were 0.73 and 0.54 in patients with normal and elevated Q_ALB_, respectively (p = 0.6, n = 34).

#### CSF lactate

**Fig 2:**
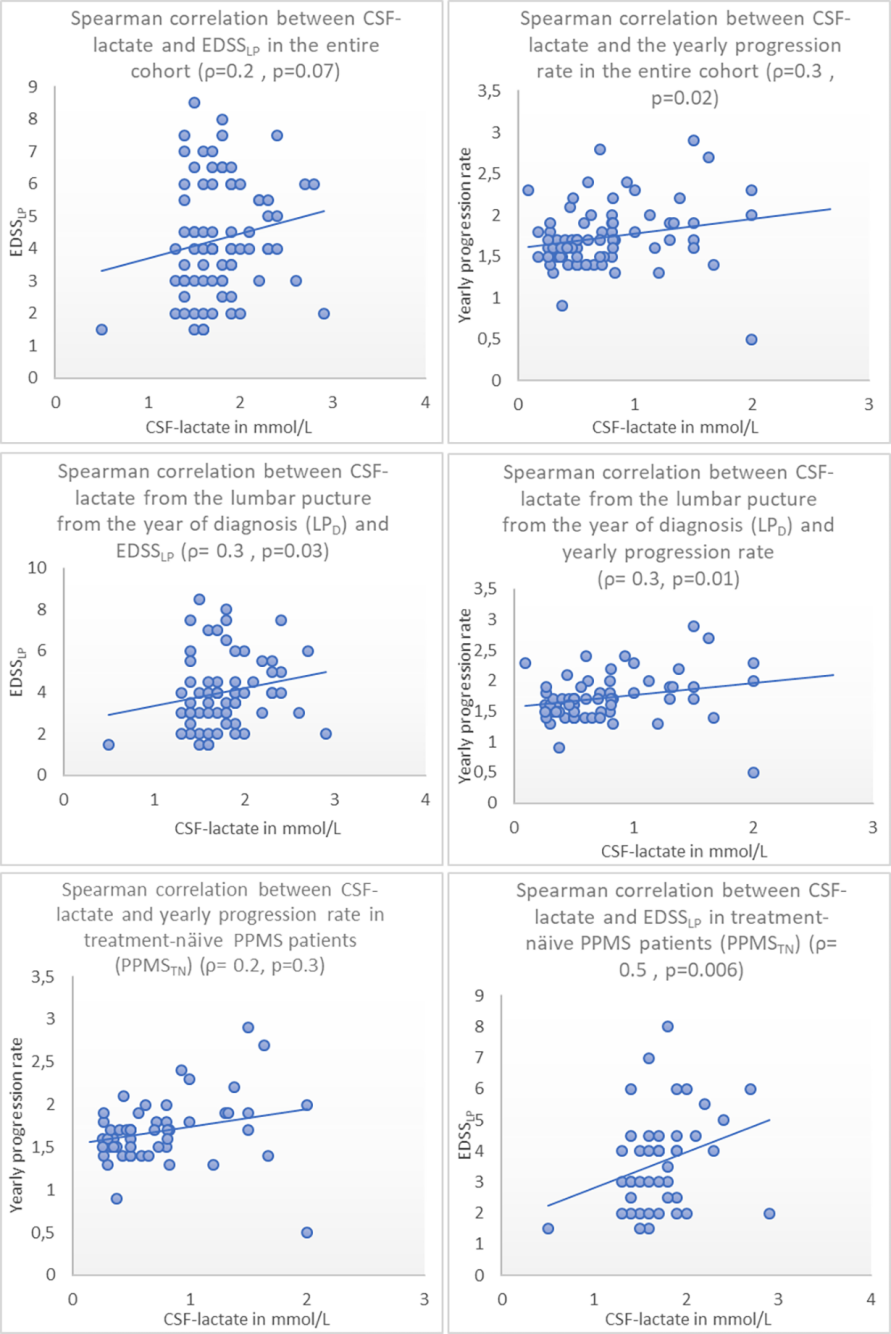
Correlation between CSF-lactate and EDSSLP and yearly progression rate in the entire cohort, EDSSLP and LPD. Spearman correlation between the CSF-lactate and expanded disability score scale at the LP (EDSS_LP_) and yearly progression rate showed consistently statistically significant positive correlation in the entire cohort, treatment-näive patients PPMS_TN_ and in the results of the diagnostic LP (LP_D_). On the other side, a significant correlation between CSF-lactate and EDSS_LP_ was found only in the LP_D_.

CSF-lactate levels were consistently correlated with the progression rate in the entire cohort, the LP_D_ and PPMS_TN_ subgroups. After excluding one case that had an extremely high lactate level (5.6 mmol/L), we found a correlation between CSF-lactate levels and EDSS_LP_ scores in the LP_D_ subgroup but not in the entire cohort or the PPMS_TN_ subgroup (see **Table 4**and **Fig 2**). CSF-lactate levels did not correlate with the patients’ age at the time of LP.

**Table 4:** Spearman correlation (ρ) between CSF-lactate and EDSS at the lumbar puncture (EDSSLP) and the yearly progression rate in the entire cohort, treatment-naïve PPMS patients (PPMSTN) and at the LP at the year of diagnosis (LPD).

	Entire cohort	PPMS_TN_	LP_D_
**Correlation between CSF-lactate in (mmol/L) and EDSS**_**LP**_	ρ = 0.2 (p = 0.07) (n = 80)	ρ = 0.2 (p = 0.3) (n = 33)	ρ = 0.3 (p = 0.03) (n = 66)
**Correlation between CSF-lactate in (mmol/L) and yearly progression rate**	ρ = 0.3 (p = 0.02) (n = 76)	ρ = 0.5 (p = 0.006) (n = 32)	ρ = 0.3 (p = 0.01) (n = 62)

#### Intrathecal synthesis of immunoglobulins

The median EDSS_LP_ and the yearly progression rate did not differ based on the OCB pattern, MRZ reaction or among patients with elevated or normal IgG, IgM, and IgA index values.

We found no correlation between IgG–production and clinical parameters in the entire cohort and those in the LP_D_ subgroup. In the PPMS_TN_ subgroup, however, quantitative markers of intrathecal IgG synthesis (IgG index and IgG_loc_) were correlated with the yearly progression rate (ρ = 0.4; p = 0.01 and 0.02, respectively; n = 35). In patients with intrathecal IgM/A synthesis, we found a moderate negative correlation between both IgM_loc_, IgA_loc_, and the yearly progression rate (ρ = -0.4, p = 0.03, n = 28, and ρ = -0.5, p = 0.01, n = 25, respectively).

## Discussion

Our study is, to the best of our knowledge, the largest CSF cohort reported in the literature thus far, with 254 PPMS patients. Motor impairment was the most frequent initial symptom, followed by cerebellar disturbances and sensory manifestations. These findings, along with the mean age and sex distribution, are typical for PPMS and consistent with other published cohorts[1, 2, 22]. The higher EDSS_LP_ rating for patients in the PPMS_motor_ group is probably due to the relatively high influence of motor symptoms on the EDSS overall[23]. The large proportion of PPMS patients under treatment that has not been approved illustrates the greatest known difficulty in treating patients with a severe progressive disease, as no approved therapies were available at the time of writing this article.

The median cell count did not deviate from the normal values, which is consistent with previous reports on other CSF cohorts with MS[24]. The BCB dysfunction was found in only one-third of the patients, which is in accordance with the hypothesis that inflammation is compartmentalized behind an intact blood-brain barrier (BBB)[25]. Other factors causing increased Alb-Q, such as reduced flow rate, were not assessed in our study.

Until recently, the clinical significance of CSF lactate in MS was not known[26]. Thus, in more than the half of our patients, CSF-lactate levels had not been routinely measured. Nevertheless, we have reported a normal CSF-lactate median in patients with PPMS[27]. Simone et al.[28] and Regenold et al.[29] reported increased levels of lactate in MS patients, while Aasly et al.[30] reported lower levels compared to healthy controls. However, all three studies did not include PPMS patients.

We have reported a weak to moderate positive correlation between CSF-lactate levels and the yearly progression rate in the entire cohort and all subgroups. A similar association was reported recently in a study with 118 RRMS patients[26]. A previous study also reported a positive correlation between serum lactate and the disease severity in all clinical subtypes of MS[31]. In other studies, CSF lactate correlated with the cell count, inflammatory and gadolinium-enhancing MRI lesions in MS patients[28, 32]. This might be explained through the mitochondrial dysfunction in progressive MS subtypes. Mitochondrial dysfunction with subsequent cellular hypoxia is especially relevant for the neurodegeneration of susceptible, chronically demyelinated axons that are commonly found in progressive MS subtypes[33]. However, this is rather speculative and prospective studies with a larger number of patients are essential to validate the prognostic value of testing CSF-lactate levels in PPMS.

The higher incidence of intrathecal IgG production indicated by OCBs compared to the elevated IgG-index and IgG_loc_ confirms the well-known higher sensitivity of isoelectric focusing (IEF)[34]. Our results were consistent with McLean et al.’s study, which reported OCB in 86–88% of samples from a cohort that included 31 progressive MS patients[9], but higher than those reported from the PROMiSe trial cohort (80%)[35]. OCBs pattern 2 was found the most frequently in our PPMS patients (79% of patients), matching the results of McLean et al.’s study[9]. In contrast, Villar et al. observed a predominance (64%) of OCBs pattern 3, but the sample size was much smaller than that of our study (n = 39)[36]. The role of systemic inflammation in the disease progression of PPMS is described elsewhere[37]^,^ [38]. In our study, we did not find any differences between yearly progression rates in patients with OCBs patterns 2 and 3. Nevertheless, studies with a larger sample size are needed to confirm our results.

In our study, the frequency of elevated IgG-index values was lower than in the cohort described by Izquierdo et al.[39]. However, Izquierdo et al.’s study included only 23 PPMS patients, who were diagnosed according to the diagnostic criteria specified by Poser. We did not find any positive correlations between EDSS_LP_ or disease progression rates and intrathecal IgG synthesis, except in the PPMS_TN_ group. However, because of the small number of patients in this group, these results should be evaluated with caution.

The MRZ reaction has been proposed as a highly specific (rule-in rather than rule-out) marker of MS[40, 41], which discriminates well between MS and neuromyelitis optica [42, 43] and possibly also between MS and MOG encephalomyelitis [44]. A positive MRZ reaction was found in in slightly more than half of the PPMS patients, a slightly lower percentage than what was observed in clinically isolated syndrome (CIS) and RRMS 60–70%[12, 41, 45] and is similar to a recent report from one of the study centres [46]. The difference might reflect lower prevalence of polyspecific immunoglobulin synthesis in PPMS or may be explained by various assays applied in different studies.

Data reports on the prevalence of intrathecal IgM synthesis in PPMS are scarce. We only found one such study, which reported elevated IgM index in 32.3% of its 29 patients with chronic progressive MS[47]. Increased IgM-index values might include false-positive results due to the linear formula used. Likewise, no studies investigating the intrathecal IgA synthesis in PPMS have been published thus far.

For the first time, a negative correlation between absolute levels of intrathecally produced IgM and IgA in CSF and disease progression has been reported in a large PPMS cohort. The correlation we found might indicate a possible protective role (e.g., anti-inflammatory or remyelinating) for IgM and IgA in PPMS. This role, postulated from *in-vitro* results, may be explained by the stimulatory effects of IgM on the oligodendrocytes as well as axonal protection[48, 49].

By using the IgX index and the Reiber formula to calculate the absolute amounts of intrathecal synthesis of immunoglobulins in mg/l, it becomes clear that the discrepancies among the prevalence of intrathecal IgG, IgM, and IgA synthesis are caused by the linear (IgX index) versus non-linear (IgX_loc_) relationships between the Q_ALB_ and Q_IgX_[19].

The main limitations of this study include the retrospective cohort design and the incomplete clinical and CSF data for some patients. However, the large number of patients in our cohort and the multicentric aspect of the design are major advantages of study, which may be the last one to include the treatment of naïve PPMS patients as different effective disease-modifying drugs are expected to enter the market in the near future [50, 51].

In summary, our study included the most detailed CSF results of the largest PPMS cohort hitherto reported, with 254 PPMS patients. The main findings were the following: a) the high diagnostic sensitivity of intrathecally produced OCBs, which mainly consisted of pattern 2; b) the positive correlation of CSF-lactate levels with clinical severity and yearly progression rates; and c) the possible protective role of intrathecally synthesized IgM and IgA, which may be of therapeutic relevance.
